# Supplementary material for: Simulated poaching affects global connectivity and efficiency in social networks of African savanna elephants—An exemplar of how human disturbance impacts group-living species
Source: PLoS Comput Biol. 2022 Jan 18;18(1):e1009792. doi: 10.1371/journal.pcbi.1009792 (PMC8797174; doi:10.1371/journal.pcbi.1009792)
Supplement: S1 Table — Detailed here are the number of clan, bond and core groups, as well as individuals per population; the number of bond and core groups, and individuals per clan; the number of core groups per group; and the number of individuals per bond and core groups. The distribution of age categories within each core group was the following: young adults (mean = 2 individuals, min = 1, max = 5); prime adults (mean = 2, min = 0, max = 7); mature adults (mean = 1, min = 0, max = 3); and matriarchs (mean = 1, min = 1, max = 1). The composition of the empirically based population is included as a reference (i.e., = 10 core groups including a total of n = 83 individuals) [80,91]. (DOCX) [file pcbi.1009792.s001.docx]

**S1 Table.** **The composition of 100 virtual population according to kinship.**

| **Demographic group** | **Minimum** | **Maximum** | **Median** | **Empirical contrast** |
| --- | --- | --- | --- | --- |
| Clan groups per population | 1 | 8 | 5 | 3 |
| Bond groups per population | 1 | 28 | 14 | 8 |
| Core groups per population | 5 | 86 | 40 | 10 |
| Bond groups per clan group | 1 | 5 | 3 | 4,3,1 |
| Core groups per clan group | 1 | 20 | 9 | 5,4,1 |
| Core groups per bond group | 11 | 5 | 3 | 1,1,2,1,2,1,1,1 |
| Individuals per population | 95 | 760 | 350 | 83 |
| Individuals per clan group | 10 | 175 | 74 | 39,36,8 |
| Individuals per bond group | 1 | 45 | 25 | 10,6,19,4,16,11,9,8 |
| Individuals per core group | 4 | 15 | 8 | 10,6,9,10,4,6,10,11,9,8 |
