# Supplementary material for: Simulated poaching affects global connectivity and efficiency in social networks of African savanna elephants—An exemplar of how human disturbance impacts group-living species
Source: PLoS Comput Biol. 2022 Jan 18;18(1):e1009792. doi: 10.1371/journal.pcbi.1009792 (PMC8797174; doi:10.1371/journal.pcbi.1009792)
Supplement: S2 Table — These statistics express the difference between targeted and random deletions in empirically based networks, along the deletion proportion axis, with deletions performed according to either age category or betweenness centrality [96]. Bold values indicate medium (≥ |0.5|) and large (≥ |0.8|) effect size. (DOCX) [file pcbi.1009792.s002.docx]

**S2 Table.** **Results of Hedge’s g test expressing the effect size difference between mean values of clustering coefficient as well as the weighted forms of modularity, diameter and global efficiency indices.**

| **Network level index** | **Deletion proportion** | **Hedge’s g statistic** | |
| --- | --- | --- | --- |
|  |  | **Age category** | **Betweenness centrality** |
| Clustering coefficient | 0.04 | -0.0410 | **-1.9865** |
|  | 0.08 | -0.0071 | **-2.8046** |
|  | 0.12 | -0.0048 | **-3.6267** |
|  | 0.16 | -0.0131 | **-4.4485** |
|  | 0.2 | -0.0548 | **-4.9924** |
| Modularity W | 0.04 | -0.0617 | -0.0643 |
|  | 0.08 | 0.0032 | 0.0202 |
|  | 0.12 | -0.1563 | -0.1106 |
|  | 0.16 | -0.0006 | -0.0960 |
|  | 0.2 | 0.1214 | -0.0314 |
| Diameter W | 0.04 | 0.2366 | **-0.7472** |
|  | 0.08 | 0.2814 | -0.3748 |
|  | 0.12 | 0.2762 | **0.5328** |
|  | 0.16 | 0.2100 | **1.9615** |
|  | 0.2 | 0.0027 | **3.9782** |
| Global efficiency W | 0.04 | 0.0028 | **-1.9351** |
|  | 0.08 | 0.0756 | **-2.8511** |
|  | 0.12 | 0.1192 | **-3.8601** |
|  | 0.16 | 0.1710 | **-4.9253** |
|  | 0.2 | 0.2101 | **-5.7467** |
