# Supplementary material for: Simulated poaching affects global connectivity and efficiency in social networks of African savanna elephants—An exemplar of how human disturbance impacts group-living species
Source: PLoS Comput Biol. 2022 Jan 18;18(1):e1009792. doi: 10.1371/journal.pcbi.1009792 (PMC8797174; doi:10.1371/journal.pcbi.1009792)
Supplement: S3 Table — The effect size differences, calculated as the Hedge’s g test, are presented as mean values for each network index in targeted and random deletions in the virtual networks, in the 500 network time step and deletion proportion increments. The deletions were performed according to age category or betweenness centrality [96]. Bold values indicate medium (≥ |0.5|) and large (≥ |0.8|) effect size. (DOCX) [file pcbi.1009792.s003.docx]

**S3 Table.** **Results of Hedge’s g test expressing the effect size difference between targeted and random deletions in virtual populations.**

| **Deletion metric** | **Network level index** | **Hedge’s g statistic per Deletion proportion** | | | | |
| --- | --- | --- | --- | --- | --- | --- |
|  |  | 0.04 | 0.08 | 0.11 | 0.16 | 0.20 |
| Age category | Clustering coefficient | **0.8433** | **1.0919** | **1.2456** | **1.3611** | **1.4424** |
|  | Modularity W | 0.0058 | -0.0265 | -0.0713 | -0.1049 | -0.1704 |
|  | Diameter W | 0.1787 | 0.1998 | 0.1652 | 0.1049 | 0.0006 |
|  | Global efficiency W | **-0.8702** | **-1.0431** | **-1.1026** | **-1.0660** | **-0.9396** |
| Betweenness  centrality | Clustering coefficient | **-1.8977** | **-1.9487** | **-1.9668** | **-1.9762** | **-1.9819** |
|  | Modularity W | 0.2267 | 0.3804 | **0.6300** | **0.8812** | **1.1268** |
|  | Diameter W | 0.1361 | 0.4427 | **0.8413** | **1.2756** | **1.6890** |
|  | Global efficiency W | **-1.3163** | **-1.6882** | **-1.8516** | **-1.9251** | **-1.9604** |
