# Supplementary material for: Simulated poaching affects global connectivity and efficiency in social networks of African savanna elephants—An exemplar of how human disturbance impacts group-living species
Source: PLoS Comput Biol. 2022 Jan 18;18(1):e1009792. doi: 10.1371/journal.pcbi.1009792 (PMC8797174; doi:10.1371/journal.pcbi.1009792)
Supplement: S4 Table — The filtering process was carried out before the onset of the deletions by dividing the value of each link in the association matrix by the highest link value and eliminating the links with values up to three percent of the highest link in increments of one percent [107]. Only 500-time step networks were considered in these experiments. (DOCX) [file pcbi.1009792.s004.docx]

**S4 Table.** **The summary of the percentages of filtered, virtual networks that broke down into two or more modules as a result of the deletions performed according to age category or betweenness centrality.**

| **Deletion metric** | **Deletion type** | **Filtering percent** | **Deletion proportion** | | | | | **Minimum, Maximum number of modules at 0.2 deletion** |
| --- | --- | --- | --- | --- | --- | --- | --- | --- |
|  |  |  | 0.04 | 0.08 | 0.12 | 0.16 | 0.2 |  |
| Age category | Targeted | 1 | 0 | 0 | 0 | 0 | 0 | 1, 1 |
|  |  | 2 | 0 | 0 | 0 | 0 | 0 | 1, 1 |
|  |  | 3 | 25.32 | 26.58 | 27.85 | 27.85 | 27.85 | 1, 2.54 |
|  | Random | 1 | 0 | 0 | 0 | 0 | 0 | 1, 1 |
|  |  | 2 | 0 | 0 | 0 | 0 | 0 | 1, 1 |
|  |  | 3 | 44.30 | 51.90 | 68.35 | 74.68 | 89.87 | 1, 2.27 |
| Betweenness centrality | Targeted | 1 | 0 | 0 | 0 | 0 | 0 | 1, 1 |
|  |  | 2 | 6 | 14 | 17 | 19 | 19 | 1, 1 |
|  |  | 3 | 63.29 | 78.48 | 86.08 | 86.08 | 86.08 | 1, 11 |
|  | Random | 1 | 0 | 0 | 0 | 0 | 0 | 1, 1 |
|  |  | 2 | 1 | 5 | 7 | 11 | 16 | 1, 1 |
|  |  | 3 | 41.77 | 56.96 | 65.82 | 78.48 | 84.81 | 1, 2.21 |
