# Supplementary material for: Simulated poaching affects global connectivity and efficiency in social networks of African savanna elephants—An exemplar of how human disturbance impacts group-living species
Source: PLoS Comput Biol. 2022 Jan 18;18(1):e1009792. doi: 10.1371/journal.pcbi.1009792 (PMC8797174; doi:10.1371/journal.pcbi.1009792)
Supplement: S1 Fig — The 500-time step cut-off was based on when the density of existing interactions among network members started to reach a plateau (~ 75% median density) [82]. The values embedded (red text) are approximated equivalents from the empirically based network prior to the beginning of the deletion experiments (Fig 4). The values for the diameter weighted can only be compare qualitatively (Figs 4 and 5). Unlike the empirically based network using association indexes in the [0,1] range, the virtual networks used the number of interactions as expression of associations. This was a consequence of the virtual network simulation process. (DOCX) [file pcbi.1009792.s005.docx]

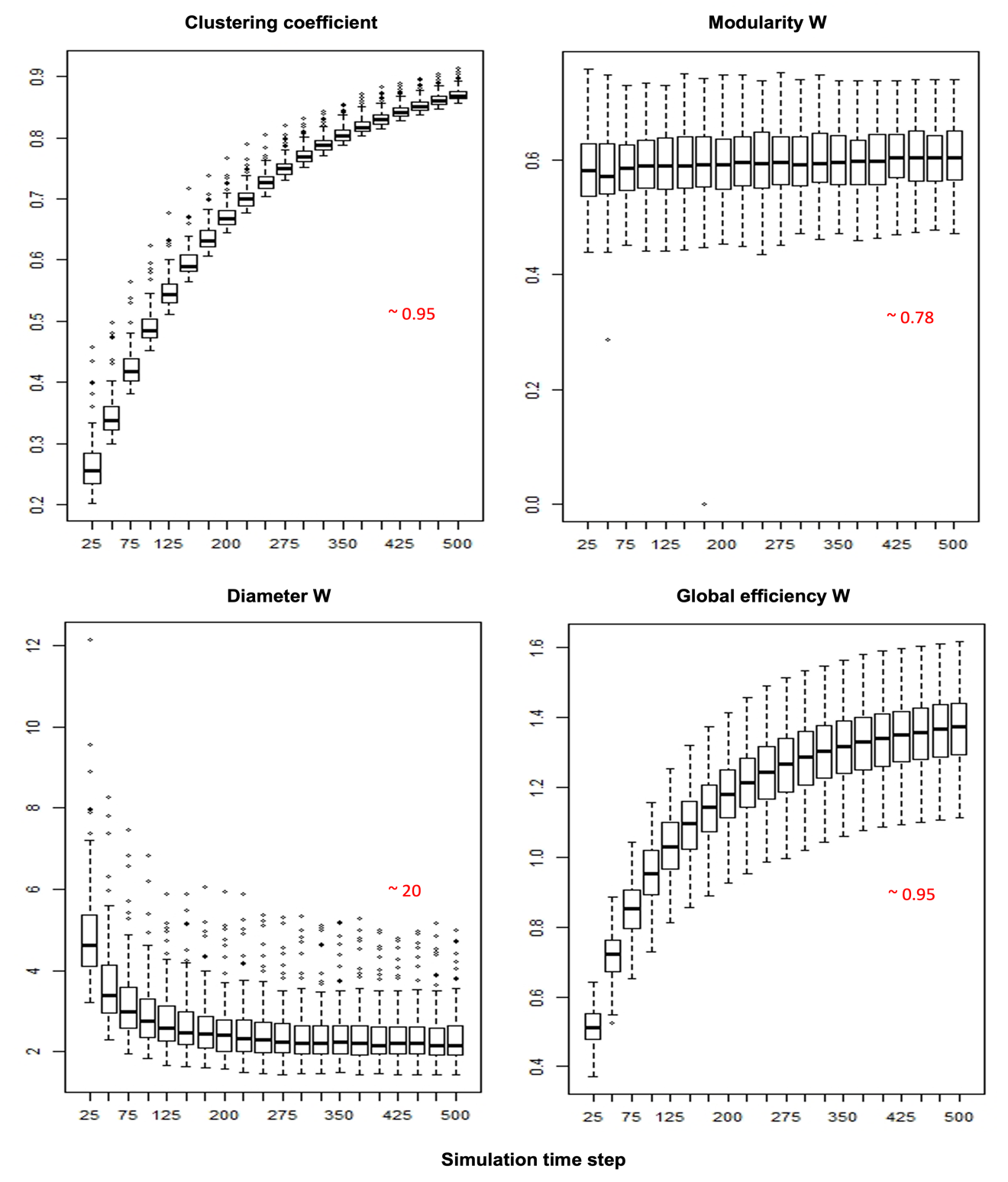


**S1 Fig.** **The distribution of values for the clustering coefficient, as well as weighted diameter, global efficiency and modularity, expressed as a function of the number of simulation time steps.**
