# Supplementary material for: Simulated poaching affects global connectivity and efficiency in social networks of African savanna elephants—An exemplar of how human disturbance impacts group-living species
Source: PLoS Comput Biol. 2022 Jan 18;18(1):e1009792. doi: 10.1371/journal.pcbi.1009792 (PMC8797174; doi:10.1371/journal.pcbi.1009792)
Supplement: S2 Fig — These associations represent links with values up to three percent of the highest link. Here, these links are presented according to age class in a dyad (Y = young adult; P = prime adult; M = mature adult; G = matriarch) and one of four social tiers. For the summary of filtering experiments showing percentages of filtered, 500-time step, virtual networks that broke down into two or more modules as a result of the deletions performed according to age category or betweenness centrality, refer to S4 Table. (DOCX) [file pcbi.1009792.s006.docx]

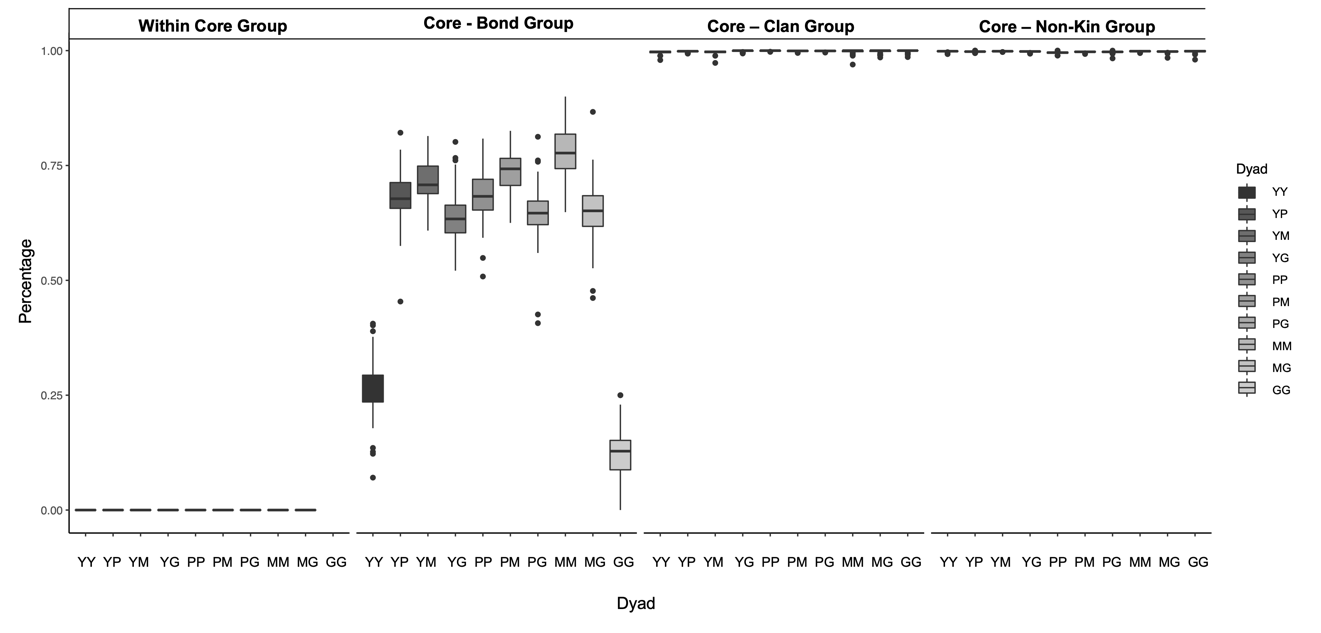


**S2 Fig.** **The percentage of the weakest associations filtered out from the 500-time step, virtual networks prior to deletion experiments.**
