# Supplementary material for: Simulated poaching affects global connectivity and efficiency in social networks of African savanna elephants—An exemplar of how human disturbance impacts group-living species
Source: PLoS Comput Biol. 2022 Jan 18;18(1):e1009792. doi: 10.1371/journal.pcbi.1009792 (PMC8797174; doi:10.1371/journal.pcbi.1009792)
Supplement: Attachment — Submitted filename: PCOMPBIOL-D-21-00628R1_comments_to_authors.pdf [file pcbi.1009792.s009.pdf]

This study concerns the effect of the removal of individuals (particularly via poaching) on the structure of animal social networks. Using African elephants as an example species, this is investigated by simulating node removal from one real-world elephant social network and an ensemble of simulated elephant social networks, and studying the effect on global network metrics. The effect of targeted removal of, respectively, older individuals and individuals with high betweenness is compared to that of random removal, and some differences are found. The study addresses an important topic, which is relevant both on a fundamental and applied level. I agree with the authors that we need a better understanding of the robustness of social networks across species, and I find the approach of investigating this by means of simulated species-specific networks interesting. I have some concerns about the methods, as well as a number of smaller suggestions. The below is rather long because I have tried to explain my thoughts clearly. I hope it will be helpful.

General comments to the methods:

1) I am not sure that the association indices (line 190-191) are calculated correctly. I believe the equation is only correct if you know with high certainty that the individuals are not together when they are both away from the group. In case you have such information, I suggest you add explanation about this to the manuscript. If you don't have such information (that is, you don't know whether they are together or not when they are away from the group), then I believe the first d needs to be removed from the equation, and the analysis should be redone using the new equation, because otherwise the association indices will be confounded and could lead to incorrect results and conclusions. The problem is that if the first d is included when you don't have information on whether they are together in these observations (when they are both away from the group), then the d just adds noise and can thereby ruin the estimates of the AIs. (Of course the d cancels out with the other d, but the same problem is still there because you end up with n instead of n-d as the denominator, meaning that you have not corrected for those group observations where you don't have information on the pair of individuals). The association index needs to measure the fraction of times they were together out of all times where you have information on their behaviour, and it seems it currently doesn't do that (unless, as mentioned, you know that they are not together when they are not in the group).

2) It is unclear to me how the equation in line 209 can be calculating what it is supposed to. I understand that the purpose of this equation is to estimate the chance that an individual i is present in its group G (which is then used further down to calculate the chance that individuals from different groups are together). The obvious way to quantify this would be to divide the number of times i was observed in group G with the total number of times G was observed (I assume that you are missing the first of these numbers since you don't do it this way but instead use dyadic data). I therefore assume that the numerator in your equation (line 209) should give an estimate of the number of times individual i was seen in group G. However, I believe that using the average here (as you do), will necessarily underestimate this number. The reason is that the true number of times individual i was in the group must be *at least* the maximum of the number of times it was observed with another individual, and the average will be lower than this. For example, if individual i was observed 2 times with individual k, 3 times with individual m, and 10 times with individual n, then it must have been in the group at least 10 times, but the average of these three numbers will be 5. This underestimating of the numerator is not a problem if it is compensated for in the denominator, but according to my checks the 'average $d_{i,j,G}$' does not make a suitable compensation for this.
Based on the above, it seems to me that it is necessary to redo the analysis with a new version of the (line 209) equation. For example, an alternative dyadic-based way, which to me seems more suitable and unbiased, could be to calculate the lower and upper bound for the possible times i could have

been in the group (based on the dyadic data), take the average of these two numbers, and then divide this by the total number of observations of the group.
It is of course also possible that I am just misunderstanding something. If this is the case then I suggest that you add explanation to the manuscript about how the equation works, so that it is clear that it is suitable for its purpose (estimating the fraction of all sightings when an individual i from core group G was seen in that group), as other readers may otherwise also wonder about this. Also, it seems a bit strange that it is not possible to count from the data the actual number of times i was observed in the group, so I would suggest adding a little explanation about this also.

3) The equation in line 219 is supposed to give the 'fraction of all sightings when group G was seen with group B. To do this, I believe the minus in the denominator should be a plus. Maybe just a typo? The description of the denominator in line 222-223 also seems incorrect.

Text-specific comments (the numbers refer to lines in the manuscript):

54-56: That's a lot of conditions for uniqueness. It seems a bit odd. I think it may work better to not use the word 'unique' and instead for example say something about 'a main contribution' of the study, or something similar.

65: Unclear what is meant by 'operate similarity'. I suppose it should have been 'similarly'?

71: The mentioning of 'interspecific' here seems out of place. Maybe it was supposed to be 'intraspecific'?

89 - : I would suggest some revision of this paragraph, such that it is clear which/whether the referenced studies are about node removal, or other types of disturbance, so that it is more obvious how they relate to your study.

89: You could consider not using the acronym, as it is easier to read without it and you only use it a few times.

93: Something seems wrong with the sentence structure, I would suggest changing 'understanding' to 'Our current understand of'.

138-142: I think this sentence needs a bit of restructuring. Currently it says (shortened version to show the structure): '…we evaluated four indices … (…); as well as the efficiency of social diffusion…(…)' , which doesn't make sense, as the social diffusion measures are part of the four indices.

149-157: I think currently it may not be entirely clear to readers how you arrive at these predictions. I think a main problem is that the reader at this stage does not know that by 'centrality' you actually mean betweenness. Many readers may be thinking of other centrality measures, such as weighted degree, and then it is not clear why removing high-centrality individuals should necessarily lead to these predictions. I suggest you mention here which kind of centrality it is you are considering, to avoid confusion, as the different centrality measures are measuring very different things. And potentially also give a little more explanation about how you arrive at the predictions.

191: Please add information about what the criterion was for 'seen together'. Was it within a certain distance of each other (and if so which), or in the same group (and if so how were groups defined)?

This is a fundamental aspect of the data and therefore important to include, so that it is clear to the reader what kind of social relationships are being measured.

203: could you give some more information about the criteria used for merging groups? Or were the bond groups defined in the referenced paper (in that case I suggest you state this directly and refer to it for details).

206: For the section starting here, I think it would be easier to follow if you presented the overall purpose and the last equation (line 227) first, and then explained how you calculate the different parts.

252-253: with the current sentence structure it is not really clear what it is that is "together" and "collectively" being referred to in the parentheses.

Table 1:

- I would suggest changing the > signs in the right column to something else. I first read them as the mathematical > signs and was a bit confused.
- I am surprised by the definition of clustering. This seems an unusual way to calculate this metric. Are you sure you calculated it in the described way (and that the given references use this definition)?
- It is generally not entirely clear from the text how you calculate the measures. I think it would help a lot if the equations you used were shown.

319: how was kinship and ages assigned to the simulated individuals? I suggest you add this information to the manuscript.

320: Why did you use a triangular distribution and how did you parametrise it? I suggest you add information about this to the manuscript.

330: I think this information about differences between the artificial and real networks is really important, because it indicates to which extent the results may correspond to those of real populations. I feel this needs some more attention. You could for example show it more clearly by showing distributions of global network measures for the artificial populations and indicate where in each distribution the measure from the real network lies.

389: not sure why it says 'simulated' here as I think this is for the empirical network?

405: I would suggest that you show a figure also for these results, as they are some of your main results and a figure will give a much better overview and also facilitate comparison with the other results.

481 - 494: But such direct application to management decisions may require a good understanding of the reactions to the disturbance. I feel this should be considered.

495-497: This statement is very general, but it could lead to fragmentation for other networks and other node removal percentages. I suggest modifying the statement.

The discussion in general: It seems that the results from the empirical network are not mentioned in the discussion. Given that you also conduct the analysis on this network, and these results are likely to give the most realistic picture, I feel it would be relevant to mention them (at least in terms of how they compare to the other results, in particular because the artificial networks differ in some aspects from the real network, as you mention in the results section).
