# Supplementary material for: Simulated poaching affects global connectivity and efficiency in social networks of African savanna elephants—An exemplar of how human disturbance impacts group-living species
Source: PLoS Comput Biol. 2022 Jan 18;18(1):e1009792. doi: 10.1371/journal.pcbi.1009792 (PMC8797174; doi:10.1371/journal.pcbi.1009792)
Supplement: Attachment — Submitted filename: Comments to authors, PCOMPBIOL-D-21-00628R2.pdf [file pcbi.1009792.s011.pdf]

I think the authors have done a good job of solving the issues I raised. I still have one main concern about the methods (the first comment below), as well as a number of smaller comments with suggestions for corrections and clarifications to the text.

The equation in line 221:

It seems to me that this equation is still not doing what it should do, and therefore is likely to give you incorrect estimations of between-group connectedness. The problem lies in using the AI's in the numerator. This is problematic because the AI of an individual depends not only on its own sociality but also that of its partners; and furthermore, it does not quantify a general tendency of socialising, but rather the chance that a specific dyad socialises (in particular because your data are only on dyadic associations and you don't have observations from when individuals were seen not associating with anyone). An example with 3 scenarios: Scenario 1): Say we have an individual i, who have been observed once, where it interacted with an individual j, who was also observed only this once. Their AI would be 1 because they were together 100% of their observations. Scenario 2) Then let's say we have an individual i, who was again observed once, but this time it was interacting with an individual who was observed twice (interacting with another individual the other time). Their AI would be 0.5, even though the behaviour of i is exactly the same as in the first scenario. Scenario 3) This time individual i has been observed 10 times, where it was interacting with the same individual all times, who was also observed only these 10 times (i.e. the same as scenario 1 but they were observed more often). They would get an AI of 1, i.e. the same as scenario 1, even though they were observed in the group much more often (and thus should be much more likely to be present when another group is encountered). Thus, it seems to me the current equation does not provide the estimate that you need, and instead gives you incorrect measures of the chance of interacting with individuals from another group. Given the new information about your data that I have got from the revision, and the description in the text, I think maybe what you actually want is: *(number of observed associations involving i) / (total number of observed associations for the group)*. Here the numerator is the raw count of i's associations (rather than AIs). The equation thus gives you the chance that an observed association between members of group G (i's group) involves individual i, which should be a good proxy for the chance that i will associate with someone from another group when group G meets another group.

Text-specific comments

150: from your description of predicted parameter changes it seems to me that it is mainly the long-range connections that are predicted to disappear (thereby increasing the modularity etc), so I think it should say 'fewer interactions with distant partners' here, rather than immediate partners?

144-152: It appears that you make the same predictions for the two cases (age and betweenness removal). This must rely on an assumption/knowledge that the older ones are the ones with higher betweenness. I would suggest mentioning this explicitly in the text (in particular because in some other systems it would be the younger ones who connect the subgroups and have high betweenness, and thus the predictions could seem surprising/odd to some readers).

160: 'platform' sounds to me like it is referring to an actual available software.

192: It is not obvious to me what is meant by ‘the main body of the group’ , and without knowing what it meant by this, the definition is obscure. I would suggest adding a bit more explanation.

209: I assume ‘group’ here refers to core group and suggest adding this to the text for clarity.

220: ‘j members’ sounds like j is a number. I assume what you mean is ‘a member (j) of its subgroup’ and suggest changing it to something like this.

312: The description of path weight is not clear to me. What is meant by the ‘length’ of a link here? I suggest you clarify in the text.

315 (Table 1):

- the (1) in the betweenness centrality description should have been (2) I think?
- the fist > in the ‘age category’ description seems to be the wrong direction.
- first line in the clustering coefficient description seem to be missing ‘closed’
- The explanation of global efficiency: There seem to be some errors in the equations. It seems that ‘shortest path’ (eq. 2 and 3) is incorrect and it should the *inverse of the length of* the shortest path? And I think you are missing a division by 2 in the denominator of eq. 3 (assuming each dyad is only included once in the numerator)?
- There seems to be some words missing from the explanation of modularity. Also, in the equations, the ‘ number of links between individuals in each dyad’ doesn’t make sense as there can only be one link between the two individuals of a dyad. I suggest revision.
- In general for table 1, I appreciate that you have added equations, but given the above described issues I suggest you go over them in detail to make sure that they do not inadvertently provide incorrect information about network index calculation. To get clear notation, it may help using i,j notation and indicating what you are summing over.

338, 344, maybe other places: ‘the probability of AI’ doesn’t make sense (as it means ‘the probability of association index’). I assume you mean ‘the probability of association’ and suggest changing the text accordingly.

393 (Figure 3): it seems that there is only links between two core groups in the empirical networks, and between none in the virtual ones. This is surprising - is it really the case? If not then I suggest redrawing the figures in a way so that the between-group links are visible.

599: This statement is still given as being for any network, but animal social networks show high structural variation, and disturbance (of the given level) is likely to fragment some networks (e.g. networks of lower density or with other structural differences). Maybe you are thinking specifically of elephants here? I would suggest either adding ‘elephants’ (e.g. in line 600, ‘…members in elephant populations’), or add that this is only for networks with structures and densities similar to the ones studied here. I think this is important because currently the statement could give the impression that poaching (of the given level) is unlikely to fragment animal populations in general (and thus have relatively minor effect), which would be misleading because the effect depends on the structure and for other networks and species poaching at the given level could have much stronger effect and fragment the networks.
