# Supplementary material for: Simulated poaching affects global connectivity and efficiency in social networks of African savanna elephants—An exemplar of how human disturbance impacts group-living species
Source: PLoS Comput Biol. 2022 Jan 18;18(1):e1009792. doi: 10.1371/journal.pcbi.1009792 (PMC8797174; doi:10.1371/journal.pcbi.1009792)
Supplement: Attachment — Submitted filename: Wisniewska et al. eleSNA MS. PlosCB. eLife Review.docx [file pcbi.1009792.s007.docx]

Editor’s global take

-simulated elephant poaching to investigate the impact of selective individual removal on the functional resilience of animal social networks to human-induced disturbance

-topic is interesting and timely, because understanding how threatened animal populations are impacted by humans is of critical importance and requires more study

-especially for species/processes with limited real-world data, but with a potentially strong impact on ecosystem functioning.

Editor’s global critique

-the **l**ogic and assumptions underlying the study are problematic and, thus, limit the insights that can be drawn from the simulation results

-Clarified the emphasis on the loss of group members with different, potentially important social roles (seniors as expectedly influential given their experience; so are younger members as they are important social players, albeit somehow less experienced); and how such a loss may be impactful on the structural characteristics of networks

-the network metrics used to infer functionality are not supported by field data on elephants, or indeed any other study systems

-Clarified that this our goal in this manuscript is not to measure cohesion/transmission directly but instead measure network indices that are commonly associated with cohesion/transmission

----------------------------------------------------------------------------------------------------------------------------------------------------------------------------

Reviewer #1 take

-Using a simulation approach, the authors investigate the impact of removing group members likely to possess key social or ecological information on the topology of elephant social networks in order to better understand how poaching pressure may influence their resilience and functionality

-Removals were based on 3 metrics thought to correlate with an individual's knowledge (age, degree, betweenness centrality) and compared to random removals for both an empirical network and virtual networks.

-Whereas targeted removals based on age had relatively limited impact on networks characteristics, removal of socially central individuals led to less integrated networks with potential consequences for the spread of adaptive information.

-The manuscript was generally clear and well-written.

-The introduction nicely laid out the rationale for this study and the authors do a nice job walking the reader through the steps of the simulation (how the networks were constructed, how deletions were performed, etc.).

-I also appreciated the discussion given to the limitations of their approach, such as the lack of network restructuring in response to removals.

Reviewer #1 critique

-Authors should be more cautious in attributing functional meaning to their network metrics, particularly given that data was unavailable to allow them to simulate a transmission process.

-Clarified that our goal is not to measure cohesion/transmission directly but instead measure indices that are commonly associated with with cohesion/transmission

-For example, at L461-463, it is stated that targeted removal of individuals with high betweenness decreased the speed of information flow, but what was actually found was that values for weighted diameter increased. Put another way, weighted diameter provides an indication of how rapidly information could potentially flow, but not whether it in fact does so. The actual dynamics of information flow are going to depend on the nature of the information and how it is transmitted among individuals

-As the authors note in the discussion (L627-640). I believe that the results should be reworded to focus more on what was actually found (i.e. changes in network metrics), with the potential functional relevance of those changes then examined in the Discussion**.**

-Clarified the results explicitly without using functional verbiage (e.g. global efficiency increased); followed with a statement of what it may mean in terms of group response in the context of elephant biology (e.g. suggests that the removal of the oldest elephants in simulated populations is less damaging to the network connectivity and efficiency than we expected)

2. Is there empirical evidence to suggest that the mature elephants that possess high-quality information are those characterized by high degree or betweenness?

-Betweenness (hubness/being bridges for interactions among others) is evidenced in elephants.

My interpretation of literature tells me that both old (Goldenberg et al 2016) and ‘very young’/juvenile (Esposito 2008) elephants are greater hubs than those of intermediate age classes. In forest eles, it is not so obvious, which is interesting [(Goldenberg et al. 2021)](https://paperpile.com/c/2gZz8s/ReSh)

Minor Comments:

L43-45: I realize this is addressed below, but please define for readers what is meant here by a 'multi-level social structure'.

-Adjusted as requested

L83-85: It would be helpful to elaborate on these previous results to provide some additional context for readers. Did herd composition change because elephants were killed or because elephants modified their behavior in poaching-heavy areas? What structural elements of their social networks remained unchanged?

-Adjusted as requested

L101-103: I don't see this as a helpful comparison with the examples below. Centrality in Rosenthal et al. reflects spatial positioning during mere seconds of interaction, rather than centrality that reflects long-term social bonds. As with the shiners, an elephant that happens to be on the outskirts of its herd might also be expected to have better access to information about its immediate environment.

-Since the manuscript narrative was reframed, this is NA

L167-170: I was glad to see this addressed here. I had been wondering about this point throughout the introduction. Would it be useful to state this even earlier (e.g., ~L135)?

-Adjusted as requested

L208: I assume everything after the / belongs in parentheses?

-Corrected

L212-214: Please modify to: "... we assembled a dyadic association matrix by combining the WCG and BCG data as described below."

-Corrected

Figure 1: The association indices for several categories appear twice-e.g., YP and PY are identical, but both are presented. Removing those duplicates would make this figure less busy.

-Corrected

Figure 3: The edge colors are very difficult to differentiate here.

-Corrected

Figure 4: Weighted modularity is missing from the y-axis.

-Corrected

Figure 6: Same comment as for Figure 1.

-Adjusted as requested (now fig 4S in supplement)

----------------------------------------------------------------------------------------------------------------------------------------------------------------------------

Reviewer #2 take

The manuscript represents a lot of hard work on an interesting topic. Understanding how threatened populations are impacted by human-derived processes is critical and requires more study.

Reviewer #2 critique

However, as it stands, the study suffers from some logical flaws that detract from the scientific insights that can be gained from this study. These are:

1. The authors argue that older individuals are important repositories of ecological knowledge, which is now well-established knowledge. However, the authors then build their study around the consequences of poaching in terms of the effects on network metrics that are assumed to correspond to transmission properties. The logical problem here is that removing ecological knowledge from a network leaves nothing to transmit-hence the transmission properties of the network are inconsequential.

2. Linked to this point is the issue that the results and discussion focus a lot on the concept of network transmission, but the study uses network metrics (e.g., diameter) as proxies of transmission properties. It is pretty well known that there are many factors (e.g., clustering coefficient) that contribute to transmission dynamics, and it is unlikely that any one network metric alone can capture the ability for a network to transmit information**.**

-Clarified that our goal is not to measure cohesion/transmission directly but instead measure indices that are commonly associated with with cohesion/transmission

I do state that we are not after measuring the degree of experience that does/does not exist in the network.

Clarified how/why we consider two indices commonly used to inform about group cohesion and other two indices commonly used to speculate about the transfer of social currency

3. The authors note that continuous data on the reorganization of the network after poaching are not existent, and that they justify using a static approach (i.e., the network does not change after a removal/simulated poaching event) by focusing on the consequences immediately after deletion. However, the simulations involve removing up to 20% of the individuals in the population, meaning that their model assumes that poaching events are occurring substantially faster than the network is reorganizing itself. This seems too unrealistic an assumption.

-The assumption here is that there is a heavy poaching pressure. We are not trying to represent real units of time

4. A further issue with using a static approach is that the networks captured in the study may not represent the network structure that is in place when an event takes place in which ecological knowledge is important. For example, studies from other multilevel societies, e.g., hamadryas baboons (from Kummer's work), suggest that units come together when conditions necessitate forming larger groups. So, the network measured in the empirical data may not be the network through which ecological knowledge is transferred when an event necessitates it.

-I address this stating that there is probably an association matrix correlation between ‘highly social’ and ‘mundane’ states. Plus, where observations are collected (waterholes), all sorts of behaviors are happening

5. Finally, the results and the conclusions drawn from the study seem in conflict. On the one hand, the main summary of the results are that removing older individuals has little, if any, impact on the network's capacity to transmit information. On the other, the conclusions seem to be slanted towards removal of older individuals as a conservation issue (e.g., L662). Thus, there is tension in the manuscript that, unfortunately, reduces both the clarity of the findings and the clarity of the take-home messages.

-Since the manuscript narrative was reframed, this is NA

Overall, the study was enjoyable to read, with lots of biology, which is a strength for a modelling study. However, some of its construction, and the reliance on simple node deletions, really limits the capacity to gain substantial new insights from this study**.**

-Our approach is common practice in the analysis of ASNA (e.g., [(Puga-Gonzalez et al. 2019)](https://paperpile.com/c/gnvwvw/01LM))

Minor Comments:

There is a tendency in the manuscript to give value to the network itself. For example, in L31 it talks about networks being subjected to stress-but the network itself isn't a biological entity.

-Clarified that the network is an emergent property of a group setting

There are also sentences that warrant citations, such as L43 and L666 as two examples

-Adjusted as requested

L46: Is there really evidence that elephants have one of 'the most complex' social systems? It doesn't seem necessary to make such strong statements.

L78: Is there evidence that older elephants hold knowledge about complex social dynamics? At the very least, this warrants some clarification (because it would be interesting).

-There is not absolute evidence that ’older elephants hold knowledge about complex social dynamics’, but decades worth of behavioral records indicate that older elephants are repositories of socioecological information (e.g., [(McComb et al. 2011)](https://paperpile.com/c/gnvwvw/Z0nz))

L120: The reduction in ecological knowledge, as suggested here, would be caused by the removal of this knowledge from the network, as opposed to any network effects per se.

-Clarified that our goal is not to measure the amount of the knowledge in the network but instead measure indices that are commonly associated with with group cohesion/transmission of social currency (with social currency assumed to exist and circulate in the network)

L133: The use of 'replicates' is a bit misleading. These are just 100 **runs** of the simulation, where (at best) one parameter se

-Adjusted as requested

L160: It is unclear if weakest links are just removed or if they are replaced by others. If the former, then it is not surprising that the removal of weakest links has a major impact on network diameter.

-Adjusted as requested

L269: Does this statement (20% of individuals have 80% of connections) hold in spatially distributed animal populations?

-Provided additional reference suggesting the importance of few nodes in organization/development of small world networks [(Rispoli et al. 2014)](https://paperpile.com/c/gnvwvw/a5vR)

L281: Heuristics does not seem to be the correct word here.

-Corrected

----------------------------------------------------------------------------------------------------------------------------------------------------------------------------

Reviewer #3 take

This manuscript attempts to address a timely question about animal social networks - what is their functional resilience to human-induced disturbance? The authors use association data from savanna elephants to construct empirical and virtual networks and assess how these change after virtual removal of individuals based on their age or network position (to simulate poaching events as real-world data were not available). Simulation studies require clear statements of caveats for interpreting the results as they only predict potential direct responses of a network and cannot account for the dynamic and indirect responses that are more likely to occur in nature. Here various network metrics are used to infer functionality, but critically, these are not supported by field data or citations (either from elephants or other study systems), and furthermore the relevance of the metrics to address structure vs. function is unclear to readers less familiar with SNA. Secondly, the motivation for the study is deeply embedded in elephant biology and would benefit a broader audience with a clear introduction to structural vs. functional resilience.

-Clarified the emphasis on the loss of group members with different, potentially important social roles (seniors as expectedly influential given their experience; so are younger members as they are important social players, albeit somehow less experienced); and how such a loss may be impactful on the structural characteristics of networks

-The idea of disturbance to a network is now introduced as a non-system specific problem

Reviewer #3 critique

1. Applicability of simulation studies

The study sets out to test the functional resilience of elephant networks after simulated poaching events because real-world data were not available (to the authors). There are many caveats for applying the results of network simulations to real-world data because they rarely can take indirect and dynamic responses into account (unless these data are used to inform the simulation), see Shizuka & Johnson Behav Ecol 2020 for a nice review of this point. The authors allude to this in the discussion when they discuss the need for more dynamic models but conclude by stating the need to work more collaboratively - this is a good point and I'm sure it's true, but there really needs to be a clear statement about the applicability of these simulated results in the introduction and upfront in the discussion. This is essential to avoid inadvertently misleading readers less familiar with these methods.

-We made clear the goals and limitations of our approach in the Abstract, Introduction, Methods, and Discussion

2. Network measures need greater empirical support and explanation

As this is a simulation exercise, it is essential that the network metrics are meaningful in this context. This is especially important given recent discussion of metric hacking in social network analysis studies (e.g., Webber et al. Anim Behav 2020). At present, some of the metrics are presented in a paragraph in the Introduction with vague support e.g., line 281 - "Each of these heuristics... SHOULD change drastically...", and all 7 are in table 1 but there are no references (either from elephants or even broadly speaking from studies on networks) to support the major assumptions of the study. Refs are given in the table caption, but it is unclear what these relate to. There have been some very interesting experimental studies on functional resilience which might help in this regard. E.g., Maldonado-Chaparro et al. 2018 PRSB used captive zebra finches to experimentally test foraging efficiency (i.e., functionality) of social groups after repeated disturbances to their networks, and as here, focused on functional change immediately after disturbance (e.g., line 172-73).

-Where appropriated, we provided additional references supporting our statements about the network indices used.

-We rephrased the description of the indices evaluated to reflect their mechanistic meaning and their biological interpretation in the context of group sociality

More importantly, it is unclear which of the 7 metrics are supposed to inform us explicitly about structure vs. function or whether these can even be unambiguously disentangled - e.g., is clustering coefficient structure or function? It is used in both this study and by Goldenberg et al. 2016 that is introduced here as focusing only on structural resilience. It would be very helpful to have clear statements about the metrics and predictions regarding structural vs. functional resilience. At the moment they vary throughout the manuscript, e.g., referred to as metrics of social competence in the discussion (line 543). Sorry for my confusion, but there are so many different ways that we can derive metrics from networks that justifying these clearly is critical for the conclusions of the study.

-Clarified the emphasis on the loss of group members with different, potentially important social roles (seniors as expectedly influential given their experience; so are younger members as they are important social players, albeit somehow less experienced); and how such a loss may be impactful on the structural characteristics of networks

-Where appropriate, we provided additional references supporting our statements about the structural characteristics of social networks and the mechanistic/biological meaning of the indices evaluated.

3. More succinct presentation of the knowledge gap and it's broader implications beyond elephant biology.

At present, the study is presented with elephant biology and conservation as the core motivation, yet the concept of functional resilience is fundamental for studies of any species where social connections influence the flow of information (and presumably fitness of individuals). The introduction is extremely long (10 paragraphs over 6.5 pages) and functional resilience is not introduced and defined until the end of the Introduction's 4th paragraph and its link to broader literature is confusing (see specific comments). Focusing the introduction on how/why structural and functional resilience may vary in networks (and how this can be inferred from network metrics), and then using elephant biology as an example for why this is relevant to study, might make it much easier to follow.

-The idea of disturbance to a network is now introduced as a non-system specific problem

Minor Comments:

Specific comments:

Abstract - the big knowledge gap being addressed here is not apparent. Stating it more clearly would make the paper more enticing to a broader readership. The results are also a little obtuse. e.g., is network connectedness a measure of structure or function?

Lines 98-114: this section appears to provide the basis for the broader rationale of the study and attempts to define functional resilience of social networks with examples. However, it ends up reading as a list of examples that lacks a cohesive statement to make it clear for readers where the knowledge gap lies. Less informed readers will need help here to see the link between these examples. Please describe the social structure of golden shiners as you do on line 104. This section reads oddly as at times the species are named and then concepts mixed with other studies on very different species. e.g., "In three tit species..." then the next sentence "Removal of such individuals" implies that this is still about the three tit species when actually it's about macaques! This is important as these are ecologically, socially, and of course taxonomically very distinct. Furthermore, as far as I'm aware, no one has demonstrated that social networks in tits are "structured around lifelong interactions" as is implied by this passage in its current form.

-Clarified our goal in the abstract (to begin addressing the link between structural features and group performance in disturbed networks) using simulated poaching experiments and quantifying network indices associated with group cohesion and transfer of social currency

Line 111-114: the meaning of this sentence is unclear. What does "Whereas" compare to? How does it link with the final clause about other species? More importantly, how does this relate to knowledge gaps about functional resilience? Please check your use of "Whereas" throughout the manuscript as each of the sentences are missing their relative clause.

-Corrected

Line 296: this is another odd use of the word "Whereas". Whereas what? The sentence is missing its comparative clause.

-Corrected

Line 443: check the wording here as it does not make sense "between the.. Versus"

-Corrected

Line 472: Figure 4 - what do the two figures displaying Modularity represent? They have the same axes labels, but different scale and the caption does not give any further information. Presumably the 2nd one is weighted?

-Corrected
