# Supplementary material for: Simulated poaching affects global connectivity and efficiency in social networks of African savanna elephants—An exemplar of how human disturbance impacts group-living species
Source: PLoS Comput Biol. 2022 Jan 18;18(1):e1009792. doi: 10.1371/journal.pcbi.1009792 (PMC8797174; doi:10.1371/journal.pcbi.1009792)
Supplement: Attachment — Submitted filename: Wisniewska et al. eleSNA MS. PlosCB. Responses to Reviewers.docx [file pcbi.1009792.s008.docx]

24 May, 2021

Dear Editorial Board at Plos Computational Biology,

Thank you for your interest in publishing our manuscript, titled ‘Simulated poaching

affects global connectivity and efficiency in social networks of African savanna elephants

— an exemplar of how human disturbance impacts group-living species~~’~~ as a research

article in journal *PLOS Computational Biology.*

On behalf of my co-authors, I would like to thank the reviewers for their time and constructive comments. Their feedback helped us improve our manuscript. In this document,

we highlight the general and specific concerns of the reviewers and our effort to address these concerns. Our responses appear in a sequence the comments were originally provided.

The content of the manuscript has changed substantially. Our responses to some comments

cannot be referenced back to the specific lines in the manuscript submitted to eLife.

Instead, we respond by annotating the sections most parallel with those in the previous version

of our manuscript.

Sincerely,

Maggie Wiśniewska, on behalf of the authors

**Reviewer #1**

-Using a simulation approach, the authors investigate the impact of removing group members likely to possess key social or ecological information on the topology of elephant social networks in order to better understand how poaching pressure may influence their resilience and functionality

-Removals were based on 3 metrics thought to correlate with an individual's knowledge (age, degree, betweenness centrality) and compared to random removals for both an empirical network and virtual networks.

-Whereas targeted removals based on age had relatively limited impact on networks characteristics, removal of socially central individuals led to less integrated networks with potential consequences for the spread of adaptive information.

-The manuscript was generally clear and well-written.

-The introduction nicely laid out the rationale for this study and the authors do a nice job walking the reader through the steps of the simulation (how the networks were constructed, how deletions were performed, etc.).

-I also appreciated the discussion given to the limitations of their approach, such as the lack of network restructuring in response to removals.

Comment 1

Authors should be more cautious in attributing functional meaning to their network metrics,

particularly given that data was unavailable to allow them to simulate a transmission process.

For example, at L461-463, it is stated that targeted removal of individuals with high

betweenness decreased the speed of information flow, but what was actually found was that

values for weighted diameter increased.

Put another way, weighted diameter provides an indication of how rapidly information could potentially flow, but not whether it in fact does so. The actual dynamics of information flow are going to depend on the nature of the information and how it is transmitted among individuals.

As the authors note in the discussion (L627-640). I believe that the results should be reworded to focus more on what was actually found (i.e. changes in network metrics), with the potential functional relevance of those changes then examined in the Discussion**.**

We clarified that our goal was not to measure cohesion/transmission directly but instead measure theoretical indicators associated with these biological features. We also clarified our results by using explicit statements about the patterns seen in the indices, instead of making statements about the patterns in the biological features themselves (e.g., global efficiency increased…).

We followed with statement of what per index patterns may suggest about group response / elephant biology

(e.g., the removal of the oldest elephants in simulated populations appeared less damaging to the network connectivity and efficiency than we expected).

We used similar language in the virtual network section below.

Comment 2

Is there empirical evidence to suggest that the mature elephants that possess high-quality

information are those characterized by high degree or betweenness?

Relative to others, and perhaps for different reasons, both senior (Goldenberg et al 2016) and

junior (Esposito 2008) elephants are social hubs.

Older individuals appear more knowledgeable (e.g., during antipredator defense McComb et al.

2011).

Comment 3

Minor Comments:

L43-45: I realize this is addressed below, but please define for readers what is meant here by a

'multi-level social structure'.

We clarified the meaning of multilevel sociality.

Comment 4

L83-85: It would be helpful to elaborate on these previous results to provide some additional

context for readers. Did herd compositions change because elephants were killed or because

elephants modified their behavior in poaching heavy areas? What structural elements of their

social networks remained unchanged?

We provide additional explanation on the subject on Goldenberg et al. 2016.

Comment 5

L101-103: I don't see this as a helpful comparison with the examples below. Centrality in

Rosenthal et al. reflects

spatial positioning during mere seconds of interaction, rather than centrality that reflects long

term social bonds. As with the shiners, an elephant that happens to be on the outskirts of its

herd might also be expected to have better access to information about its immediate

environment.

This reference was removed from the revised manuscript.

The highlighted paragraph is relevant in terms of introducing the knowledge gap and why it

should be addressed.

Comment 6

L167-170: I was glad to see this addressed here. I had been wondering about this point

throughout the introduction.

Would it be useful to state this even earlier (e.g., ~L135)?

Throughout the sections of the manuscript, we provide the statement about the limitations of our

approach, starting in the abstract and here in the introduction. We also discuss our results in

the context of these limitations.

Comment 7

L208: I assume everything after the / belongs in parentheses?

We corrected the sign omission.

Comment 8

L212-214: Please modify to: "... we assembled a dyadic association matrix by combining the

WCG and BCG data as described below."

We corrected this sentence as recommended.

Comment 9

Figure 1: The association indices for several categories appear twice-e.g., YP and PY are

identical, but both are presented. Removing those duplicates would make this figure less busy.

We corrected the figure labels as suggested.

Comment 10

Figure 3: The edge colors are very difficult to differentiate here.

We modified the figure by adding color bar for clarification of meaning of color coded edges.

Comment 11

Figure 4: Weighted modularity is missing from the y-axis.

We corrected the omissions as recommended (now Fig 3).

Comment 12

Figure 6: Same comment as for Figure 1.

We adjusted the labeling as requested (now fig S3 in the supplement).

**Reviewer #2**

The manuscript represents a lot of hard work on an interesting topic. Understanding how

threatened populations are impacted by human-derived processes is critical and requires more

study.

However, as it stands, the study suffers from some logical flaws that detract from the scientific

insights that can be gained from this study. These are:

Comment 13

1. The authors argue that older individuals are important repositories of ecological knowledge,

which is now well established knowledge. However, the authors then build their study around

the consequences of poaching in terms of the effects on network metrics that are assumed to

correspond to transmission properties. The logical problem here is that removing ecological

knowledge from a network leaves nothing to transmit-hence the transmission properties of

the network are inconsequential.

2. Linked to this point is the issue that the results and discussion focus a lot on the concept of

network transmission, but the study uses network metrics (e.g., diameter) as proxies of

transmission properties. It is pretty well known that there are many factors (e.g., clustering

coefficient) that contribute to transmission dynamics, and it is unlikely that any one network

metric alone can capture the ability for a network to transmit information**.**

We clarified that individuals of various ages may be socially influential (although perhaps differently; seniors – more ecological experience; juniors -gregariousness) and that removal of seniors is unlikely to leave the networks without any socially valuable properties

currency. We also clarified that our goal is not to measure cohesion/transmission directly but instead measure theoretical indices* commonly associated with these biological features.

*two indices commonly used to inform views about group cohesion (clustering coefficient, modularity), and other two indices to inform about the capacity for transfer of social currency (diameter, global efficiency)

Comment 14

3. The authors note that continuous data on the reorganization of the network after poaching are

not existent, and that they justify using a static approach (i.e., the network does not change after

a removal/simulated poaching event) by focusing on the consequences immediately after

deletion. However, the simulations involve removing up to 20% of the individuals in the

population, meaning that their model assumes that poaching events are occurring substantially

faster than the network is reorganizing itself. This seems too unrealistic an assumption.

We clarified that our goal was to characterize the effect of different levels of poaching pressure, ranging from relatively low to intensive. We also clarified that, given our design, we cannot account for the absolute amount of time passing between poaching events.

Comment 15

4. A further issue with using a static approach is that the networks captured in the study may not

represent the network structure that is in place when an event takes place in which ecological

knowledge is important. For example, studies from other multilevel societies, e.g., hamadryas

baboons (from Kummer's work), suggest that units come together when conditions necessitate

forming larger groups. So, the network measured in the empirical data may not be the network

through which ecological knowledge is transferred when an event necessitates it.

We clarified that there is likely a notable parallel in network structure between ‘highly social’ states referenced to by the reviewer and the states observed near waterholes/vantage points* in the empirical data used for parametrization.

Comment 16

5. Finally, the results and the conclusions drawn from the study seem in conflict. On the one

hand, the main summary of the results are that removing older individuals has little, if any,

impact on the network's capacity to transmit information. On the other, the conclusions seem to

be slanted towards removal of older individuals as a conservation issue (e.g., L662). Thus, there

is tension in the manuscript that, unfortunately, reduces both the clarity of the findings and the clarity of the take-home messages.

In the revised manuscript, we give equal attention to both deletion metrics, instead of overfocusing on the age-specific deletions. In the discussion, we reasoned through both sets of results in the context of elephant biology/sociality. The discussion of the implications of our work for conservation are written keeping in mind the limitations of ours simulation approach.

Comment 17

Overall, the study was enjoyable to read, with lots of biology, which is a strength for a modelling

study. However, some of its construction, and the reliance on simple node deletions, really limits

the capacity to gain substantial new insights from this study**.**

Our approach follows established methods in the analysis of ASNA (e.g., [(Puga-Gonzalez et al.2019)](https://paperpile.com/c/gnvwvw/01LM). Although this is not a novel method approach, evaluation of network indices

associated with not only connectivity (as done in one previous study) but also the theoretical

capacity for transfer of social currency in a large # of elephant-like populations (versus 1 preciously evaluated population ) is novel.

Comment 18

There is a tendency in the manuscript to give value to the network itself. For example, in L31 it

talks about networks being subjected to stress-but the network itself isn't a biological entity.

We clarified that the network is an emergent property of a group setting.

Comment 19

There are also sentences that warrant citations, such as L43 and L666 as two examples

We provided pertinent resources in this section as well as in the adjusted as requested in

relevant section (lines 477 -84).

Comment 20

L46: Is there really evidence that elephants have one of 'the most complex' social systems? It

doesn't seem necessary to make such strong statements.

L78: Is there evidence that older elephants hold knowledge about complex social dynamics? At

the very least, this warrants some clarification (because it would be interesting).

Although there is perhaps not explicit evidence that ‘older elephants hold knowledge about complex social dynamics’, decades of behavioral records indicate that elephants have a complex form of sociality and that older elephants are repositories of socioecological information and recognize more conspecifics than junior counterparts (e.g., among others, (McComb et al. 2011- shows importance of the older matriarch and tests their knowledge; Goldenberg et al. 2017 -shows the importance of the experienced individuals in formatting of social bonds by their offspring and how their disappearance stymies this process).

Comment 21

L120: The reduction in ecological knowledge, as suggested here, would be caused by the

removal of this knowledge from the network, as opposed to any network effects per se.

We clarified that our goal is not to measure the amount of the knowledge in the network, but instead to measure associated theoretical indices. As explained in our response to comment 13 (related), we assumed that some form of social currency exists and circulates in the network before as well as after poaching of seniors.

* two indices commonly used to inform views about group cohesion (clustering coefficient, modularity), and other two

indices to inform about the capacity for transfer of social currency (diameter, global efficiency)

Comment 22

L133: The use of 'replicates' is a bit misleading. These are just 100 **runs** of the simulation,

where (at best) one parameter se

We adjusted the wording as suggested.

Comment 23

L160: It is unclear if weakest links are just removed or if they are replaced by others. If th

former, then it is not surprising that the removal of weakest links has a major impact on network

diameter.

We clarified this statement as requested.

Comment 24

L269: Does this statement (20% of individuals have 80% of connections) hold in spatially

distributed animal populations?

We provided an additional reference suggesting the importance of few nodes in organization/development of small world, biological networks [(Rispoli et al. 2014)](https://paperpile.com/c/gnvwvw/a5vR).

Comment 25

L281: Heuristics does not seem to be the correct word here.

We rephrased the relevant section as suggested.

-------------------------------------------------------------------------------------------------------------------------------

**Reviewer #3 summary**

Comment 26

This manuscript attempts to address a timely question about animal social networks - what is

their functional resilience to human-induced disturbance? The authors use association data

from savanna elephants to construct empirical and virtual networks and assess how these

change after virtual removal of individuals based on their age or network position (to simulate poaching events as real-world data were not available). Simulation studies require clear

statements of caveats for interpreting the results as they only predict potential direct responses

of a network and cannot account for the dynamic and indirect responses that are more likely to

occur in nature. Here various network metrics are used to infer functionality, but critically, these

are not supported by field data or citations (either from elephants or other study systems), and

furthermore the relevance of the metrics to address structure vs. function is unclear to readers

less familiar with SNA. Secondly, the motivation for the study is deeply embedded in elephant

biology and would benefit a broader audience with a clear introduction to structural vs.functional

resilience.

We clarified the emphasis on the loss of group members with different/potentially important social roles (seniors - experience; juniors -gregariousness) and how such losses may be impacting the structural characteristics of networks. It is not our goal to tackle questions about functional consequences of network disturbance (our model is not designed to directly test hypotheses about functional effects of disturbances/ e.g., lower fitness). The idea of disturbance to a network is presented as a non-system specific problem.

Comment 27

1. Applicability of simulation studies

The study sets out to test the functional resilience of elephant networks after simulated

poaching events because real world data were not available (to the authors). There are many

caveats for applying the results of network simulations to real-world data because they rarely

can take indirect and dynamic responses into account (unless these data are used to inform the

simulation), see Shizuka & Johnson Behav Ecol 2020 for a nice review of this point. The authors

allude to this in the discussion when they discuss the need for more dynamic models but

conclude by stating the need to work more collaboratively - this is a good point and I'm sure it's

true, but there really needs to be a clear statement about the applicability of these simulated

results in the introduction and upfront in the discussion. This is essential to avoid inadvertently

misleading readers less familiar with these methods.

We clarified the goals and limitations of our approach in the Abstract, Introduction, Methods, and Discussion sections.

Comment 28

2. Network measures need greater empirical support and explanation

As this is a simulation exercise, it is essential that the network metrics are meaningful in this

context. This is especially important given recent discussion of metric hacking in social network

analysis studies (e.g., Webber et al. Anim Behav 2020). At present, some of the metrics are

presented in a paragraph in the Introduction with vague support e.g., line 281 - "Each of these

heuristics... SHOULD change drastically...", and all 7 are in table 1 but there are no references

(either from elephants or even broadly speaking from studies on networks) to support the major

assumptions of the study. Refs are given in the table caption, but it is unclear what these relate

to. There have been some very interesting experimental studies on functional resilience which

might help in this regard. E.g., Maldonado-Chaparro et al. 2018 PRSB used captive zebra

finches to experimentally test foraging efficiency (i.e., functionality) of social groups

after repeated disturbances to their networks, and as here, focused on functional change

immediately after disturbance (e.g., line 172-73).

We provided additional explanation and references (in text and Table 1) supporting our statements about network indices used (Lines 95-103; 252-69).

We rephrased the description of the indices used to reflect their theoretical meaning and a potential biological interpretation.

Comment 29

More importantly, it is unclear which of the 7 metrics are supposed to inform us explicitly about

structure vs. function or whether these can even be unambiguously disentangled - e.g., is

clustering coefficient structure or function? It is used in both this study and by Goldenberg et al.

2016 that is introduced here as focusing only on structural resilience.

It would be very helpful to have clear statements about the metrics and predictions regarding

structural vs. functional resilience. At the moment they vary throughout the manuscript, e.g.,

referred to as metrics of social competence in the discussion (line 543). Sorry for my confusion,

but there are so many different ways that we can derive metrics from networks that justifying

these clearly is critical for the conclusions of the study.

We clarified our emphasis on the loss of group members with different, potentially important social roles (seniors as well as juniors) and how such a loss may impact the structural characteristics of the networks (instead of functional characteristics as our modeling approach is not designed to test hypotheses about functional properties of a network).

Relative to the previous version of the manuscript, we presented and discussed a reduced number of indices to quantify network structure (1. connectedness and 2. partitioning; as well as 3. diameter and 4. theoretical efficiency of connections allowing for a flow of social currency). We made sure to use consistent terminology regarding

our interest in evaluating presence /absence of socially influential individuals (not solely defined

by age).

Comment 30

3. More succinct presentation of the knowledge gap and it's broader implications beyon

elephant biology.

At present, the study is presented with elephant biology and conservation as the core

motivation, yet the concept of functional resilience is fundamental for studies of any species

where social connections influence the flow of information (and presumably fitness of

individuals). The introduction is extremely long (10 paragraphs over 6.5 pages) and functional

resilience is not introduced and defined until the end of the Introduction's 4th paragraph and its

link to broader literature is confusing (see specific comments). Focusing the introduction on

how/why structural and functional resilience may vary in networks (and how this can be inferred

from network metrics), and then using elephant biology as an example for why this is relevant to

study, might make it much easier to follow.

We introduced the idea of disturbance to a network is as a non-system specific problem and

shortened the introduction.

Comment 31

Abstract - the big knowledge gap being addressed here is not apparent. Stating it more clearly would make the paper more enticing to a broader readership. The results are also a little obtuse. e.g., is network connectedness a measure of structure or function?

In the abstract, we clarified the problem – assessing network structure, specifically the

theoretical indicators of group performance/resilience in disturbed networks.

In the results, we described findings focusing on network structure and offered a perspective on

potential biological meaning.

Comment 32

Lines 98-114: this section appears to provide the basis for the broader rationale of the study and

attempts to define functional resilience of social networks with examples. However, it ends up

reading as a list of examples that lacks a cohesive statement to make it clear for readers where

the knowledge gap lies. Less informed readers will need help here to see the link between these

examples. Please describe the social structure of golden shiners as you do on line 104. This

section reads oddly as at times the species are named and then concepts mixed with other

studies on very different species. e.g., "In three tit species..." then the next sentence "Removal

of such individuals" implies that this is still about the three tit species when actually it's about

macaques! This is important as these are ecologically, socially, and of course taxonomically

very distinct. Furthermore, as far as I'm aware, no one has demonstrated that social networks in

tits are "structured around lifelong interactions" as is implied by this passage in its current form.

The abovementioned references are no longer a part of the revised manuscript.

This paragraph (lines 89-103) provides the broad rational and relevant references leading to the specific example where we consider how perturbing the structural features in a simulated social network changes its theoreticalcapacity for maintaining a well-connected and efficient system for potential exchange of social currency.

Comment 33

Line 111-114: the meaning of this sentence is unclear. What does "Whereas" compare to? How

does it link with the final clause about other species? More importantly, how does this relate to

knowledge gaps about functional resilience? Please check your use of "Whereas" throughout

the manuscript as each of the sentences are missing their relative clause.

Comment 34

Line 296: this is another odd use of the word "Whereas". Whereas what? The sentence is missing its comparative clause.

Comment 35

Line 443: check the wording here as it does not make sense "between the.. Versus"

Response to Comments 33,4,5

In the revised manuscript, we stated that our focus is on the structural effects of poaching on simulated, elephant social networks, and that we quantified the theoretical indicators of network performance/resilience (instead of considering them as empirical metrics). We made clear that we were not testing how the structural changes affect the functioning of elephant groups in an absolute sense (e.g., Line 120-1).

We corrected the use of ‘whereas’ where appropriate (line #s referenced above are no longer relevant).

Comment 36

Line 472: Figure 4 - what do the two figures displaying Modularity represent? They have the

same axes labels, but different scale and the caption does not give any further information.

Presumably the 2nd one is weighted?

We corrected the omissions as recommended (now Fig 3).
