# Supplementary material for: Simulated poaching affects global connectivity and efficiency in social networks of African savanna elephants—An exemplar of how human disturbance impacts group-living species
Source: PLoS Comput Biol. 2022 Jan 18;18(1):e1009792. doi: 10.1371/journal.pcbi.1009792 (PMC8797174; doi:10.1371/journal.pcbi.1009792)
Supplement: Attachment — Submitted filename: renamed_7afba.docx [file pcbi.1009792.s010.docx]

6 September 2021

Dear Editorial Board at PLOS Computational Biology,

Thank you for your interest in publishing our revised manuscript, titled ‘Simulated poaching

affects global connectivity and efficiency in social networks of African savanna elephants

 — an exemplar of how human disturbance impacts group-living species~~’~~ as research

article in journal *PLOS Computational Biology.*

On behalf of my co-authors, I would like to thank the reviewers for their thoughtful comments and useful suggestions. Their feedback helped us improve our manuscript. In this document,

we detail the concerns of the reviewers and our approach to address these concerns. Our responses appear in a sequence the comments were originally provided and in the annotated version of the manuscript for easy reference.

The content of the manuscript has partly changed. Our responses to some comments

cannot be referenced back to the specific lines in the original manuscript. Instead, we respond by annotating the sections most parallel with those in the previous version of our manuscript. We recommend that the reviewers read over the annotated document for ease of reference.

Sincerely,

Maggie Wiśniewska, on behalf of the authors

Blue - the reviewers’ comments verbatim
Black - our responses to one, or multiple, comments in order as they appear in the review, or grouped together if related (e.g., comment #1 and 16 below have a common response)

**Reviewer #1**

**Comment # 1:** The within-group association index seems questionable to me. The current formula is x/(x + d + (n-d-x)) = x/n, but this is not correct. I think what you want is x/(n-d), which is the portion of group sightings when at least one of the pairs was present in which they associated.

**Comment # 16**

1) I am not sure that the association indices (line 190-191) are calculated correctly. I believe the equation is only correct if you know with high certainty that the individuals are not together when they are both away from the group. In case you have such information, I suggest you add an explanation about this to the manuscript. If you don’t have such information (that is, you don’t know whether they are together or not when they are away from the group), then I believe the first d needs to be removed from the equation, and the analysis should be redone using the new equation, because otherwise the association indices will be confounded and could lead to incorrect results and conclusions. The problem is that if the first d is included when you don’t have information on whether they are together in these observations (when they are both away from the group), then the d just adds noise and can thereby ruin the estimates of the AIs. (Of course the d cancels out with the other d, but the same problem is still there because you end up with n instead of n-d as the denominator, meaning that you have not corrected for those group observations where you don’t have information on the pair of individuals). The association index needs to measure the fraction of times they were together out of all times where you have information on their behaviour, and it seems it currently doesn’t do that (unless, as mentioned, you know that they are not together when they are not in the group).

**Response Comment # 1and 16:** We recalculated all AIs using the suggested formula correction (x/(n-d)); and rerun all simulations based on the new AIs.

**Comment # 2**

I'm not totally clear on the between group calculations. Here, the average number of days, across all partners, where individuals associated (n_ijG) or neither member was seen (d_ijG) is taken to be approximately equal to the number of days that individual was seen or the number of days the group was seen without the individual, respectively. I'm not sure if this is correct, and I'd like to see some justification of this approach. I am not sure why the authors didn't directly use data on individual occurrences, and directly took the portion of sightings of a given group in which each group member was seen. Was this a limitation of data formatting or recording (i.e. were only associations recorded, rather than individual presence)?

**Comment # 3**.

My main questions and comments have to do with the simulation approach. First, I worry that your approach may ignore individual or group level heterogeneity in social position and gregariousness. Since you assign probabilities at the dyadic level (either dyads of individuals or groups), you may lose any variation in association rates across individuals. Put another way, we might expect that associations between dyads are not independent; dyads that involve the same individual, say dyads A-B and A-C, are expected to be correlated, since they both involve A and A might have some kind of consistent sociality/gregariousness. That all gets lost when you assign dyadic association probabilities independently.

This may seem kind of pedantic and unimportant, but I don't think it is. So much of the effect you're looking for in your deletions comes from the fact that individual-level differences in social position can have big consequences for social network structure. If your simulations remove that individual-level heterogeneity, then you've lost the structure that you're actually most interested in. This could be doubly important here as you could have variation in behaviour at two levels, group and individual. I think you could either come up with a simulation framework that captures this (maybe based on fitting a multi membership mixed effects model to the association indices from the empirical data), show that individual-level variation isn't important in your network, or include some very heavy caveats to your simulation analysis.

**Comment # 17**

It is unclear to me how the equation in line 209 can be calculating what it is supposed to. I understand that the purpose of this equation is to estimate the chance that an individual i is present in its group G (which is then used further down to calculate the chance that individuals from different groups are together). The obvious way to quantify this would be to divide the number of times i was observed in group G with the total number of times G was observed (I assume that you are missing the first of these numbers since you don’t do it this way but instead use dyadic data). I therefore assume that the numerator in your equation (line 209) should give an estimate of the number of times individual i was seen in group G. However, I believe that using the average here (as you do), will necessarily underestimate this number. The reason is that the true number of times an individual i was in the group must be *at least* the maximum of the number of times it was observed with another individual, and the average will be lower than this. For example, if individual i was observed 2 times with individual k, 3 times with individual m, and 10 times with individual n, then it must have been in the group at least 10 times, but the average of these three numbers will be 5. This underestimation of the numerator is not a problem if it is compensated for in the denominator, but according to my checks the ‘average di,j,G’ does not make a suitable compensation for this.

Based on the above, it seems to me that it is necessary to redo the analysis with a new version of the (line 209) equation. For example, an alternative dyadic-based way, which to me seems more suitable and unbiased, could be to calculate the lower and upper bound for the possible times i could have been in the group (based on the dyadic data), take the average of these two numbers, and then divide this by the total number of observations of the group.

It is of course also possible that I am just misunderstanding something. If this is the case then I suggest that you add explanation to the manuscript about how the equation works, so that it is clear that it is suitable for its purpose (estimating the fraction of all sightings when an individual i from core group G was seen in that group), as other readers may otherwise also wonder about this. Also, it seems a bit strange that it is not possible to count from the data the actual number of times i was observed in the group, so I would suggest adding a little explanation about this also.

**Response  to Comments # 2, 3 and 17**

During the original data collection (spanning a period of ~ 4 years), the authors inferred proximity-based associations at two social tiers:

1) among individuals within 10 separate core groups (within core group - WCG), but group composition per each WCG sampling event was not reported in the original publication. Consequently, although we knew when individuals *i* and *j* from core group *F* were observed associating during one sampling event, we could not be certain if individual *j* was present when conspecifics *i* and *k* were observed associating during another sampling event. This prevented the determination of the dependence of associations between individuals *i* and *j* as a function of their interactions with individual *k*.

and 2) between 64 core groups (between core group - BCG), where each group was treated as a single social entity with no information about between-individual interactions was recorded.

The challenges we faced using these two datasets are the following:

Although we could not calculate a true metric of  individual gregariousness, we calculated overall values of individual association (or proxies of gregariousness) by using Eq. 1 shown below.

Eq. 1. *f_i,G_* or *f_a,B_*   =  sum (x/(n-d))/# dyads or freq. of being in a core group AND 'interacting' with

      (any/all) core group members

**Comment # 4**

I'd also generally like to see a bit more work on how well your simulations capture the structure of your empirical network. How similar are they in terms of average path length, modularity, clustering coefficient, etc.? The plots are nice, and you do indicate that the empirical networks differ from the simulated one in terms of between group association and betweenness distribution, but some general quantitative comparison would be beneficial. My main concern is that factors you didn't simulate, like the heterogeneity I mentioned above, or transitivity in network ties, may make these simulated networks fundamentally different from the observed structure of the empirical network. Things like differences in overall density are not a huge issue (since that could be due to finite sample size in the observed network), but if there are processes happening in the real social associations that aren't captured by your simulation this could be an issue.

**Comment #33**

330: I think this information about differences between the artificial and real networks is really important, because it indicates to which extent the results may correspond to those of real populations. I feel this needs some more attention. You could for example show it more clearly by showing distributions of global network measures for the artificial populations and indicate where in each distribution the measure from the real network lies.

**Response to Comment # 4 and 33**

We provided a comparison of global network indices for the empirically based network and the virtual networks in the Supplemental figure 1S.

**Comment # 5.**

What was the biological relevance of the different numbers of timesteps used? Each time step seems to, more or less, represent a day (assuming daily sampling periods for the association index). Presumably we don't think these individuals are only able to associate with one another for 100 days, and then suddenly 20% of the population is removed. I think more relevant is to use association probabilities as your "true" network. It is also entirely possible that I've just really missed something here.e

**Response to Comment # 5**

The simulation process should not be interpreted in terms of absolute time passage because the empirical association data, used as a basis for social network simulations, were collected over nearly four year; and do not contain information about when each dyadic or between-group association record was collected.

Since we did not know how many interactions would result in virtual social networks similar in their global properties to the empirically based network, we observed the virtual network development over multiple steps and stopped the simulations when the networks began platoeing.

**Comment # 6.**

Finally I also wonder about the **biological relevance of the removal of weak links**. What process in the elephant system could cause this? Habitat fragmentation, changes in resource availability, maybe behavioural changes from exploitation? I think it's a fine bit of analysis to do but putting it in the context of the system would be great.

**Response to Comment # 6**

Removal of weak links may be comparable to a poaching scenario where instead of only conspicuous seniors (given their relatively large tusks ), others are also targeted by poaching (e.g., militarized, and nearly nonrandom, version of poaching common in 2009-2016 phase).

**Comment # 7**

L 42: What kind of desirable traits are you discussing? Economically desirable?

**Response to**  **Comment #** 7

Yes. We corrected the text as suggested.

**Comment # 8**

L 50: What do you mean by "broke down"? Do you mean fractured into multiple components?

**Response to**  **Comment** # 8

Yes. We corrected the text as suggested.

**Comment # 9.**

L 85: Social brokers don't necessarily need to be more gregarious, they just need to associate with diverse individuals

**Response to Comment  # 9**

Yes, but in this case we purposefully emphasize the value of social brokers as based on their gregariousness (common in younger animals) as potentially opposed to experience; this is to bring the reader's attention to the fact that non-random harvest does not mean one thing, and can target individuals of different age category/experience/social importance. Each of such targeted deletions may impact animal social networks in a different but consequential way.

**Comment # 10**

L 107: I think matrilinear here should be "matrilineal"

**Response to  Comment # 10**

Yes. We corrected the text as suggested [Ln. 96].

**Comment # 11.**

 L 147: I might eliminate this last sentence in the paragraph

**Response to Comment  # 11**

We believe the last sentence clarifies the uniformity of experimental treatment across the empirically based and virtual networks. For that reason, we decided to retain this statement.

**Comment # 12.**

L 164: I think delimiations should be "limitations".

**Response to  Comment # 12**

Yes. We corrected the text as suggested.

**Comment # 13.**

L 232: It's not necessarily surprising that degree and betweenness are correlated; Previous simulation studies have used a metric called "cutpoint potential", the residuals from a regression of betweenness on degree, to find highly important nodes. Could this be added or at least mentioned?

**Response to  Comment # 13**

Yes. We corrected the text as suggested [(Makagon et al. 2012)](https://paperpile.com/c/G0XLRu/INlJ)

**Comment # 14.**

Table 1: "The inverse of the network's global efficiency" I think should be "The inverse of the networks average path length" or something similar. Currently it reads that efficiency is the inverse of efficiency.

**Response to Comment # 14**

Yes. We corrected the text [now it states that Weighted GE - inverse of the network’s average path length (a.k.a. global efficiency) divided by the ideal global efficiency [(Ek et al. 2015)](https://paperpile.com/c/G0XLRu/ICod).

**Comment # 15.**

Line 321: "dad" should be "dyad"

**Response to  Comment # 15**

Yes. We corrected the text as suggested .

**Reviewer #2**

**Comment # 18**

3) The equation in line 219 is supposed to give the ‘fraction of all sightings when group G was seen with group B. To do this, I believe the minus in the denominator should be a plus. Maybe just a typo? The description of the denominator in line 222-223 also seems incorrect.

**Response to  Comment # 1**8

We corrected the formula to now : *f_G,B_* = *n_G,B_* / (*n_G_* + *n_B_* + *n_G,B_*) (instead of : *f_G,B_* = *n_G,B_* / (*n_G_* + *n_B_* – *n_G,B_*)) to reflect the fraction of times when they were seen together over the total of when they were seen either together or separately? The between-family association proxies were recalculated and re-simulated using the corrected formula.

**Comment # 19**

54-56: That’s a lot of conditions for uniqueness. It seems a bit odd. I think it may work better to not use the word ‘unique’ and instead for example say something about ‘a main contribution’ of the study, or something similar.

**Response to  Comment # 19**

Yes. We corrected the text as suggested.

**Comment # 20**

65: Unclear what is meant by ‘operate similarity’. I suppose it should have been ‘similarly’?

**Response to  Comment # 20**

Yes. We corrected the text as suggested..

**Comment #21**

71: The mentioning of ‘interspecific’ here seems out of place. Maybe it was supposed to be ‘intraspecific’?

**Response to Comment # 21**

Yes. We corrected the text as suggested.

**Comment # 22**

89 - : I would suggest some revision of this paragraph, such that it is clear which/whether the referenced studies are about node removal, or other types of disturbance, so that it is more obvious how they relate to your study.

**Response to Comment # 22**

We clarified the effect of disturbance in simulated vs. biological example populations.

**Comment # 23**

89: You could consider not using the acronym, as it is easier to read without it and you only use it a few times.

**Response to Comment # 23**

Yes. We corrected the text as suggested..

**Comment # 24**

93: Something seems wrong with the sentence structure, I would suggest changing ‘understanding’ to ‘Our current understanding of’.

Response to **Comment # 24**

Yes. We corrected the text as suggested.

**Comment # 25**

138-142: I think this sentence needs a bit of restructuring. Currently it says (shortened version to show the structure): ‘…we evaluated four indices … (…); as well as the efficiency of social diffusion…(…)’ , which doesn’t make sense, as the social diffusion measures are part of the four indices.

**Response to Comment # 25**

We rephrase this sentence for clarity.

**Comment** # 26

149-157: I think currently it may not be entirely clear to readers how you arrive at these predictions. I think a main problem is that the reader at this stage does not know that by ‘centrality’ you actually mean betweenness. Many readers may be thinking of other centrality measures, such as weighted degree, and then it is not clear why removing high-centrality individuals should necessarily lead to these predictions. I suggest you mention here which kind of centrality it is you are considering, to avoid confusion, as the different centrality measures are measuring very different things. And potentially also give a little more explanation about how you arrive at the predictions.

**Response to Comment  # 26**

We clarified that it is betweenness centrality that we used to define topological centrality.

**Comment # 27**

191: Please add information about what the criterion was for ‘seen together’. Was it within a certain distance of each other (and if so which), or in the same group (and if so how were groups defined)?

This is a fundamental aspect of the data and therefore important to include, so that it is clear to the reader what kind of social relationships are being measured.

**Response to Comment # 27**

We added the requested details.

**Comment # 28**

203: could you give some more information about the criteria used for merging groups? Or were the bond groups defined in the referenced paper (in that case I suggest you state this directly and refer to it for details).

**Response to Comment # 28**

As stated at the end of this paragraph, the core groups were assigned to the same bond/clan according to genetically determined relatedness indices (presented in the original paper) and long-term, behavioral associations inferred by the authors. For clarity we stated that information about group-level  genetic relatedness can be found in the original publication.

**Comment # 29**

206: For the section starting here, I think it would be easier to follow if you presented the overall purpose and the last equation (line 227) first, and then explained how you calculate the different parts.

**Response to Comment # 29**

We restructure the section as suggested.

**Comment # 30**

252-253: with the current sentence structure it is not really clear what it is that is “together” and “collectively” being referred to in the parentheses.

Table 1:

- I would suggest changing the > signs in the right column to something else. I first read them as the mathematical > signs and was a bit confused.

- I am surprised by the definition of clustering. This seems an unusual way to calculate this metric. Are you sure you calculated it in the described way (and that the given references use this definition)?

- It is generally not entirely clear from the text how you calculate the measures. I think it would help a lot if the equations you used were shown.

**Response to Comment # 30**

-We rephrased to clarify the meaning of deletion metric/type proportion.

Table 1.

-We added detail explaining how to interpret the notations(< or >)  used to express our predictions.

-We corrected and added detail explaining how each index was calculated

**Comment # 31**

L 319: how was kinship and ages assigned to the simulated individuals? I suggest you add this information to the manuscript.

**Response to Comment # 31**

By kinship we mean membership in a given core, bond or clan. For instance, all individuals in core group EA were considered as close kin; individuals in bond groups EA and EB were considered as moderately related kin, and so forth. Kin assignment was modeled on the original paper. Please see Response to 28 for related details . Before ‘poaching experiments’, we generated a number of populations that resemble the empirical population in age category and kinship makeup.

**Comment # 32**

320: Why did you use a triangular distribution and how did you parametrise it? I suggest you add information about this to the manuscript.

**Response to Comment  # 32**

We used a triangular distribution because we do not know how the distribution of AIs actually looked, but we had data on the min and max values observed. The lower and upper end of the triangle is the lowest and highest probability observed and the peak is the median value.

**Comment # 34**

389: not sure why it says ‘simulated’ here as I think this is for the empirical network?

**Response to Comment #34**

We changed the phrase ‘simulated’ to empirically based (Ln. 478), but it is important to keep in mind that because the true networks can only be constructed at the WCG tier; our representation of the social network at the BCG level is strictly speaking a proxy (Please, see the data limitations describe on our response to # 2, 3 and # 17).

**Comment # 35**

405: I would suggest that you show a figure also for these results, as they are some of your main results and a figure will give a much better overview and also facilitate comparison with the other results.

**Response to Comment #35**

We added Fg 5 as requested.

**Comment # 36**

481 - 494: But such direct application to management decisions may require a good understanding of the reactions to the disturbance. I feel this should be considered.

**Response to Comment #36**

We clarified that the basic condition for the utility of deletion simulations (such as presented in our work) will depend on existing baseline understanding about the species-specific reactions to the disturbance  of interest. Lions are a good example of such a species for reasons described here.

**Comment # 37**

-495-497: This statement is very general, but it could lead to fragmentation for other networks and other node removal percentages. I suggest modifying the statement.

-The discussion in general: It seems that the results from the empirical network are not mentioned in the discussion. Given that you also conduct the analysis on this network, and these results are likely to give the most realistic picture, I feel it would be relevant to mention them (at least in terms of how they compare to the other results, in particular because the artificial networks differ in some aspects from the real network, as you mention in the results section).

**Response to Comment # 37**

-We added a statement that these findings hold within the range of simulated disturbance intensity tested in this research, … ‘finding the hypothetical limitations to social resilience may require evaluating more intensive yet biologically meaningful ‘poaching’ disturbance than considered in our work (117)’.

-The results for the deletions in empirically based and virtual social networks were

qualitatively similar, and for that reason we discuss them together.  Whenever appropriate, we specify the similarity or difference between the empirically based and virtual networks.
