# Supplementary material for: Simulated poaching affects global connectivity and efficiency in social networks of African savanna elephants—An exemplar of how human disturbance impacts group-living species
Source: PLoS Comput Biol. 2022 Jan 18;18(1):e1009792. doi: 10.1371/journal.pcbi.1009792 (PMC8797174; doi:10.1371/journal.pcbi.1009792)
Supplement: Attachment — Submitted filename: renamed_8f239.docx [file pcbi.1009792.s012.docx]

15 December 2021

Dear Editorial Board at PLOS Computational Biology,

Thank you for your continued interest in publishing our manuscript, titled ‘Simulated poaching

affects global connectivity and efficiency in social networks of African savanna elephants

— an exemplar of how human disturbance impacts group-living species’ as research

article in your journal.

On behalf of my co-authors, I would like to thank the reviewer for very constructive comments. The reviewer’s feedback helped us improve our manuscript. In this document, we highlight the general and specific concerns of the reviewer and our effort to address these concerns. Our responses appear in a sequence in which the comments were originally provided.

Although the content of the revised manuscript has not changed substantially, some of our

responses cannot be traced back to specific lines pointed out by the reviewer. However, we

hope that the annotated version of the revised manuscript will make the tracking of the

changes convenient.

Kind Regards,

Maggie Wiśniewska, on behalf of the authors

Reviewer’s Comment #1a

It seems to me that this equation is still not doing what it should do, and therefore is likely to give you incorrect estimations of between-group connectedness. This is problematic because the AI of an individual depends not only on its own sociality but also that of its partners; and furthermore, it does not quantify a general tendency of socialising, but rather the chance that a specific dyad socialises (in particular because your data are only on dyadic associations and you don􀍛t have observations from when individuals were seen not associating with anyone). An example with 3 scenarios: Scenario 1): Say we have an individual i, who have been observed once, where it interacted with an individual j, who was also observed only this once. Their AI would be 1 because they were together 100% of their observations. Scenario 2) Then let’s say we have an individual i, who was again observed once, but this time it was interacting with an individual who was observed twice (interacting with another individual the other time). Their AI would be 0.5, even though the behaviour of i is exactly the same as in the first scenario. Scenario 3) This time individual i has been observed 10 times, where it was interacting with the same individual all times, who was also observed only these 10 times (i.e. the same as scenario 1 but they were observed more often). They would get an AI of 1, i.e. the same as scenario 1, even though they were observed in the group much more often (and thus should be much more likely to be present when another group is encountered). Thus, it seems to me the current equation does not provide the estimate that you need, and instead gives you incorrect measures of the chance of interacting with individuals from another group. Given the new information about your data that I have got from the revision, and the description in the text, I think maybe what you actually want is: (number of observed associations involving i) / (total number of observed associations for the group). Here the numerator is the raw (rather than AIs). The equation thus gives you the chance that an observed association proxy for the chance that i will associate with someone from another group when group G meets another group.

Reviewer’s Comment #1b

Reply to the authors

Firstly, in the document you seem to calculate within-group link weights. Just for clarification, the suggestion was only meant for the calculation of the between-group link weights, where you don’t have the information to calculate AIs in the usual way. (I assume the within-group link weights are calculated directly with the AI equation in the manuscript).

My suggestion concerned the fi,G equation. I see in the document that the suggestion gives low values. But it seems to me that this is also what would be expected for between-group interactions, if individuals spend relatively little time in their group.

For example, if individual i from group A and individual j from group B each spend only 20% of time in their respective groups, then the chance of them meeting when the groups meet should be correctly estimated by 0.2*0.2, as far as I can see. I think this also still needs to be timed with fG,B (as you do in the manuscript with the p(iG , aB) equation), to take into account the fraction of time the groups spend together. This means, for example, that two individuals from different groups that are always present in their groups get a link weight that corresponds to the fraction of their time the groups spend together (the fG,B), which seems reasonable, and individuals from different groups that are less often present in their groups get links with lower weights. So, to summarize, it seems to me that the suggestion still makes logical sense and should give useful estimates of between-group link weights, using the limited information you have (but it is as always entirely possible that I am overlooking something).

A note concerning comparison to AIs: Given your data, it may not be possible to calculate something that is directly comparable to AIs for the between-group case. It seems that you will in all cases end up with a network where link weights are estimated in two different ways, and just have to take that into account the evaluation of the results. Also, the AIs (the blue numbers in your document) seem higher than what I commonly see (may again be due to the data structure where I think for example you don’t have observations of single individuals), so that may also contribute to a magnitude discrepancy.

To answer your question of whether you should proceed with modifications: assuming that the issues with the version of the fi,G equation in the manuscript that I described in my earlier comment are valid, that method does not give useful estimates (because it does not actually give the fraction of sightings in which i interacts). Therefore my suggestion would still be to redo the calculations of between-group link weights in some way or other, because link weights that don’t resemble the real-world could in the worst case lead to incorrect conclusions. To me it seems that the best way to go would be to logically deduce an equation that gives the best estimate of dyads from different groups associating, given the data available. For example what is described above (if this seems reasonable), or something else that you find makes logical sense. To me this seems a better procedure than adjusting the equations until the output looks right as you try with the U in the document.

Those are my thoughts. I will leave it to the authors to judge to which degree the above is useful and proceed in the way they find best.

Our Response to #1b

**Our response to Comment # 1 is mostly attempting to address the most recent feedback we received from the reviewer (labeled above as Comment #1b). The summary of our response to Comment #1a, which the reviewer had seen and responded to, is places at the end of our response for the convenience of the reviewer and the editor.**

Indeed, the within-group link weights are calculated with equation # 1 in the manuscript.

However, to estimate link weights at the population level (e.g., between individuals *i* from group *G* and *a* form group *B*), we cannot use the recommended approach (i.e., 0.2*iG* *0.2*aB*, where the #s indicate the proportion of time each individual spent/associated in its core group). It is because the data collected while observing within-core-groups interactions (WCG) do not contain per sampling event group composition. Consequently, even though we know that individuals *iG* and *jG* associated 20 times (out of 100 times when core group G was observed), we cannot be certain if individual *kG* (who interacted with *iG* 10 times and with *jG* 30 times) was present during the 20 WCG sampling events pertaining to *iG*-*jG* associations.

In lieu of the proportion of time each individual spent/associated in its own core group, we decided to calculate a proxy of individual gregariousness using the equation # 2 or (*f_i,G_ = ∑(AI_iG, jG_, AI_iG, kG,_ AI_iG, zG,_ AI_iG, pG …_) / # of dyads*)*.* This proxy (necessary to eventually estimate link weights at the population level) is determined for every individual in the context of its group (not between groups which seems to be a source of confusion)*.*

This proxy was next combined with the equation # 3 or (*f_G,B_ = n_G,B_ / (n_G_ + n_B_ + n_G,B_*) which determines the fraction of the sightings when the core groups *G* and *B* were seen together*.*

After combining these parameters, as indeed recommended by the reviewer in the latest correspondence, in equation # 4 or (*p(iG , aB) = f_iG_ × f_aB_ × f_G,B_*.), we estimated a proxy of dyadic association between any two individuals from different core groups.

In sum, given the limitations of our data, we believe that equations 2, 3 and 4 together lead to a most reasonable representation of a) the proportion of time each individual spent/associated in its core group; b) estimation of the link weights at the population level (in place of so called 0.2*iG* *0.2*aB* calculation); and c) ultimately help us to simulate a set of virtual populations that in our judgement resemble not only the empirically based population but also real world elephant populations much more closely than would the process highlighted in red above (and our attempts to modify it below).

Summary of Our Response to #1a

Upon reception of this comment we, run a series of diagnostics implementing the Reviewer’s suggestions (in red above) above. Our goal was to ensure that the recommended changes would recover the empirically based dyadic AI ranges, which we believe to be a reasonable manifestation of social dynamics in real elephant populations (e.g., youngest and oldest individuals are more likely to interact with or be sought after as associates than those in intermediate age classes).

Implementation of the formula (emphasized in red above) leads to a large much smaller within-core group AIs relative to the AIs calculated using empirical data.

This, in turn, results to an underestimation of the distribution of dyadic AI per age class category (red box plots – ‘New Index’) versus when using the empirical data (turquoise box plots – ‘Old Index’), which are the basis for virtual network simulation.

As a reasonable alternative (in our opinion), we modified the Reviewer’s suggestion using the follows formula:

proportion of time individual *i* spent in the core group *G* or *PTCG _iG_ = max # of observed associations per individual i / total # of core group G sighting*.

This modification let to either an underestimate (left panel below) or overestimate**(right panel below) of the distribution of dyadic AI per age class category relative to the trends seen in the empirical data.

** To minimize the differences between the recalculated AIs and the empirical based AIs, we added a constant value (U) to the proportion of time every individual was observed in the group. This value was chosen to minimize the sum of the squared differences between recalculated AI and empirical based AIs - analogous to the residual sum of squares (RSS) according to the following formula: (*PTCG _iG_ * U) ** (*PTCG _aB_ * U)*

Reviewer’s Comment #2

150: from your description of predicted parameter changes it seems to me that it is mainly the long-range connections that are predicted to disappear (thereby increasing the modularity etc), so I think it should say ‘fewer interactions with distant partners here, rather than immediate partners?’

Our Response to #2

We corrected the text as recommended emphasizing the targeted deletions are likely to decrease in richness of interactions between intermediate and distant kin.

Reviewer’s Comment #3

144-152: It appears that you make the same predictions for the two cases (age and betweenness removal). This must rely on an assumption/knowledge that the older ones are the ones with higher betweenness. I would suggest mentioning this explicitly in the text (in particular because in some other systems it would be the younger ones who connect the subgroups and have high betweenness, and thus the predictions could seem surprising/odd to some readers).

Our Response to #3

We made it clear (sentence starting on Ln 100-102) that older elephants are considered both socioecologically experienced and well connected (Goldenberg et al. 2016). This does not negate our statement about the most immature members also considered as highly gregarious.

Reviewer’s Comment #4

160: ‘platform’ sounds to me like it is referring to an actual available software.

Our Response to #4

To rid of the confusion, we changed the phrasing from ‘platform’ to ‘approach.’

Reviewer’s Comment #5:

192: It is not obvious to me what is meant by ‘the main body of the group’ , and without knowing what it meant by this, the definition is obscure. I would suggest adding a bit more explanation.

Our Response to #5:

This phrase was taken directly from the original publication (Archie et al. 2006).

To clarify, we state that the definition of a core group and its diameter were visually assessed on the basis of the group’s membership and activity synchrony among its members. This is a common technique of assessing group membership in field studies of well described elephant populations.

Reviewer’s Comment #6

209: I assume ‘group’ here refers to core group and suggest adding this to the text for clarity.

Our Response to #6

We added the clarification as suggested.

Reviewer Comment #7

220: ‘j members’ sounds like j is a number. I assume what you mean is ‘a member (*j*) of its subgroup’ and suggest changing it to something like this.

Our Response to #7

We clarify this statement by using symbols *j* and k to denote social partners of I for whom the gregariousness score is being calculated.

To be more explicit, we mean the following:

Sum of( Ais involving individual *i*)/total number of observed associations involving individual *i*.

In other words, the numerator is a sum of:

AI between *i* and *z*,

AI between *i* and *k*, and other group members if needed.

The denominator is # 2 because i in this example was seen in a dyadic association with two social partners.

Reviewer’s Comment #8

The description of the path weight is not clear to me. What is meant by the ‘length’ of a link here? I suggest you clarify in the text.

Our Response to #8

As suggested we clarified the process of defining path length.

Reviewer’s Comment #9a

315 (Table 1):

- the (1) in the betweenness centrality description should have been (2) I think?

Our Response to #9a

We shifted the subscript numbers; now the text is consistent with the suggestion.

Reviewer’s Comment #9b

the fist > in the ‘age category’ description seems to be the wrong direction.

Our Response to #9b

We corrected the symbol as recommended.

Reviewer’s Comment #9c

first line in the clustering coefficient description seem to be missing ‘closed’

Our Response to #9c

As recommended, we added the missing phrase.

Reviewer’s Comment #9d

The explanation of global efficiency: There seem to be some errors in the equations. It seems that ‘shortest path’ (eq. 2 and 3) is incorrect and it should the *inverse of the length of* the shortest path? And I think you are missing a division by 2 in the denominator of eq. 3 (assuming each dyad is only included once in the numerator)?

Our Response to #9d

As recommended, we clarify our process. Namely, we stress that first assumes that the higher the weight between two nodes, the shorter the distance between them. To reflect this relationship, we define the length of a link as the inverse of its weight. Using this inverse of the weights, we calculate all shortest paths in the network. The distance between nodes *i* and *j* is defined as the sum of the link lengths over the shortest path connecting *i* and *j*. The efficiency of ‘communication’ from *i* to *j* is then assumed to be inversely proportional to the shortest path length, 1/*dij*. When there is no path linking *i* to *j* we have dij = +infinity, and the efficiency in the ‘communication between *i* and *j* is set equal to 0. The global efficiency of graph G is then defined as the average of the efficiency in the communication over all couples of nodes:

$$GE=\frac{1}{n(n-1)}\sum_{\begin{aligned} i,j\in G \\ i\neq j \end{aligned}} \frac{1}{d_{i,j}}$$

This process is inspired by Migliano et al. 2017.

Reviewer’s Comment #9e

There seems to be some words missing from the explanation of modularity. Also, in the equations, the ‘ number of links between individuals in each dyad’ doesn’t make sense as there can only be one link between the two individuals of a dyad. I suggest revision.

Our Response to #9e

As recommended, we clarify that the consideration of the number of links occurs in the context of one (or many) module(s) in a weighted network. We also explain that we used the igraph library function cluster_leading_eigen to calculate modularity in a weighted network. In this table, we present modularity formula for the simples case (where all nodes belong to a single module) and refer the reader to the publication that is the basis for the igraph function. (Newman 2006).

Reviewer’s Comment #9f

## In general for table 1, I appreciate that you have added equations, but given the above described issues I suggest you go over them in detail to make sure that they do not inadvertently provide incorrect information about network index calculation. To get clear notation, it may help using i,j notation and indicating what you are summing over.

Our Response to #9f

As recommended, we use the recommended notations when appropriate.

# Reviewer’s Comment #10

## 338, 344, maybe other places: ‘the probability of AI’ doesn’t make sense (as it means ‘the probability of association index’). I assume you mean ‘the probability of association’ and suggest changing the text accordingly.

Our Response to #10

We corrected the phrasing as recommended in both (and only) instances.

# Reviewer Comment #11

## 393 (Figure 3): it seems that there is only links between two core groups in the empirical networks, and between none in the virtual ones. This is surprising - is it really the case? If not then I suggest redrawing the figures in a way so that the between-group links are visible.

# Our Response to #11

There are several between core group links in the virtual population. The thickness of the lines expresses link strength. We reformatted the figure to better illustrate the presence/location of both weak and strong links.

# Reviewer Comment #12

## 599: This statement is still given as being for any network, but animal social networks show high structural variation, and disturbance (of the given level) is likely to fragment some networks (e.g. networks of lower density or with other structural differences). Maybe you are thinking specifically of elephants here? I would suggest either adding ‘elephants’ (e.g. in line 600, ‘…members in elephant populations’), or add that this is only for networks with structures and densities similar to the ones studied here. I think this is important because currently the statement could give the impression that poaching (of the given level) is unlikely to fragment animal populations in general (and thus have relatively minor effect), which would be misleading because the effect depends on the structure and for other networks and species poaching at the given level could have much stronger effect and fragment the networks.

# Our Response to #12

We clarify our statement as recommended by mentioning that our findings are most applicable in the context of elephant biology and networks with similar architecture to the ones tested in our work.
